# Women feel more attractive before ovulation: evidence from a large-scale online diary study

**DOI:** 10.1017/ehs.2021.44

**Published:** 2021-09-01

**Authors:** Lara Schleifenbaum, Julie C. Driebe, Tanja M. Gerlach, Lars Penke, Ruben C. Arslan

**Affiliations:** 1 Georg August University, Goettingen, Germany; 2 Leibniz ScienceCampus Primate Cognition, Goettingen, Germany; 3 University of Leipzig, Leipzig, Germany; 4 Max Planck Institute for Human Development, Berlin, Germany

**Keywords:** ovulatory cycle shifts, self-perception, attractiveness, hormonal contraception, diary study, evolutionary psychology

## Abstract

How attractive we find ourselves decides who we target as potential partners and influences our reproductive fitness. Self-perceptions on women's fertile days could be particularly important. However, results on how self-perceived attractiveness changes across women's ovulatory cycles are inconsistent and research has seldomly assessed multiple attractiveness-related constructs simultaneously. Here, we give an overview of ovulatory cycle shifts in self-perceived attractiveness, sexual desirability, grooming, self-esteem and positive mood. We addressed previous methodological shortcomings by conducting a large, preregistered online diary study of 872 women (580 naturally cycling) across 70 consecutive days, applying several robustness analyses and comparing naturally cycling women with women using hormonal contraceptives. As expected, we found robust evidence for ovulatory increases in self-perceived attractiveness and sexual desirability in naturally cycling women. Unexpectedly, we found moderately robust evidence for smaller ovulatory increases in self-esteem and positive mood. Although grooming showed an ovulatory increase descriptively, the effect was small, failed to reach our strict significance level of .01 and was not robust to model variations. We discuss how these results could follow an ovulatory increase in sexual motivation while calling for more theoretical and causally informative research to uncover the nature of ovulatory cycle shifts in the future.

## Graphical abstract

**Figure:**
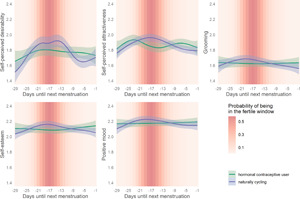

**Social media summary:** Women report higher attractiveness, desirability, self-esteem and positive mood but not more grooming when fertile.

## Introduction

There is an ongoing debate about whether the fertile phase in a woman's ovulatory cycle warrants being called an oestrus, a phase of fertility which is typically characterised by heightened sexual proceptivity, receptivity and attractiveness (Beach, 1976; Gangestad & Thornhill, 2008). Alongside other aspects such as increased sexual motivation when fertile that might indicate an oestrus-like phase (Arslan, Schilling et al., 2018; Jones et al., 2018; Roney & Simmons, 2013), it appears that women's attractiveness increases around ovulation as a possible cue to fertility (Haselton & Gildersleeve, 2011). Some studies find that various aspects of attractiveness change along with cyclical hormonal fluctuations, including body scent (Gildersleeve et al., 2012; Singh & Bronstad, 2001), vocal pitch (Pipitone & Gallup, 2008; Puts et al., 2013) and facial attractiveness (Puts et al., 2013; Roberts et al., 2004). While studies largely report that men rate women's attractiveness as higher around ovulation (Bobst & Lobmaier, 2012; Haselton & Gildersleeve, 2011; Roberts et al., 2004; Schwarz & Hassebrauck, 2008), it remains unclear whether women's self-perceived attractiveness follows the same pattern.

Since self-perceptions can guide mating decisions (Penke et al., 2008), they are relevant from an evolutionary perspective on human behaviour: within human mating markets that are characterised by mutual partner choice and assortative mating (Johnstone et al., 1996; Robinson et al., 2017), individuals are expected to calibrate their mating decisions (i.e. mating goals and mating tactics) according to their self-perceived mate value in order to avoid costs (e.g. wasted mating efforts or lost opportunities in finding other mates). Humans face trade-offs regarding different mate qualities (e.g. regarding preferred condition and attachment of partners), and one's own self-perceptions can guide the necessary degree of these trade-offs (Penke et al., 2008), meaning that individuals who deem themselves as highly valuable mates strive for higher quality partners, where less trade-offs of preferences are needed. The most relevant component of women's mate value is their physical attractiveness (Buss & Shackelford, 2008; Singh, 2002) since it is assumed to be an indicator of their youth and reproductive value (Bovet, 2019; Lassek & Gaulin, 2019). Consequently, it has been shown that women adjust their mate choices according to their self-perceived attractiveness, with women who perceive themselves as more attractive showing higher mate choice standards and choosiness, at least in short-term contexts (Little et al., 2001; Todd et al., 2007, but see Gerlach et al. (2019) for a null finding on moderation of mate preferences and actual long-term mate choice). Hence, understanding how women's self-perceived attractiveness changes across the cycle is crucial, particularly during the fertile window when conception is possible and mating decisions have a direct impact on reproductive fitness.

Using diary study designs that track within-subject changes in self-reported thoughts and behaviours over the ovulatory cycle, several studies have investigated ovulatory cycle shifts in self-perceived attractiveness but yielded mixed results: Haselton and Gangestad (2006) first presented empirical evidence in 38 heterosexual and naturally cycling women who provided daily self-reports for 35 days. These women felt both more attractive and more sexually desirable when they were fertile compared with other days of their cycles. However, Schwarz and Hassebrauck (2008) did not replicate these results using a diary design across 31 days. Analysing data from 40 naturally cycling women and comparing high- with low-fertility days, they did not find increases in self-perceived attractiveness around ovulation. In a preregistered, highly powered online diary study across 40 days using over 26,000 diary entries from 1054 women, Arslan, Schilling et al. (2018) applied a quasi-control group design that compared women taking hormonal contraceptives (625 HC women) with naturally cycling women (429 NC women). They found a robust increase in self-perceived sexual desirability that was absent in HC women. These results were supported by a wide range of robustness analyses, for example comparing different fertility estimates. Arguably, this study provides the best evidence to date that self-perceived sexual desirability indeed increases around ovulation. Since HC women do not experience ovulation and a corresponding fertile phase, the finding that cycle shifts in sexual desirability were only present in NC women supported the claim that these shifts are related to hormonal fluctuations across the natural ovulatory cycle.

As shown here, a distinction of attractiveness and sexual desirability is difficult and evolutionary psychologists often use the terms interchangeably (Wade, 2000). Addressing this issue, Wade (2000) showed that, for women, perceptions of their own attractiveness are based on their self-perceived figure, eyes and sex appeal. While their perceptions of their sexual desirability were based on their figure as well, they were also predicted by their self-perceived physical strength and sexual motivation, and less by their facial features. Whereas more research is needed to replicate these results, it seems that attractiveness and sexual desirability are closely related constructs that differ mainly in their association with sexual activity.

Owing to our limited understanding of ovulatory changes in self-perceived attractiveness and sexual desirability, the aim of the current study was not only to investigate these potential ovulatory shifts but also to investigate other closely related self-perceptions. Firstly, some studies report that women change their grooming behaviour and clothing style to appear more attractive around ovulation, possibly to attract more potential sexual partners as a form of intrasexual competition (Durante et al., 2008; Haselton et al., 2007). In a study comparing photographs taken during the high- and low-fertility phases of the ovulatory cycles of 30 partnered women, Haselton et al. (2007) found that women attempt to look more attractive when fertile. Using a similar design, but also asking women to draw illustrations of their outfits when invited to attend an imaginary social event, Durante et al. (2008) showed that 88 women wore and wanted to wear sexier clothing on high-fertility days. Other diary studies also report that women spend more time grooming when they are fertile (Röder et al., 2009; Saad & Stenstrom, 2012).

Yet, diary studies that assessed self-perceptions in grooming and attractiveness concurrently reached opposing conclusions. Whereas Röder et al. (2009) found ovulatory increases in both variables, Schwarz and Hassebrauck (2008) reported ovulatory increases only with more provocative clothing choices, and the highly powered study by Arslan, Schilling et al. (2018) only found ovulatory increases in self-perceived desirability. While grooming effort can potentially explain ovulatory increases in attractiveness ratings by men, evidence for ovulatory increases in self-perceived grooming is mixed and it remains unclear whether they co-occur with changes in self-perceived attractiveness and self-perceived desirability.

Secondly, it has been shown that feeling attractive and desirable is positively related to general self-esteem in women (Bale & Archer, 2013; Brase & Guy, 2004; Leary & Baumeister, 2000). However, past research indicates no significant ovulatory changes (Arslan, Schilling et al., 2018) or even ovulatory decreases (Hill & Durante, 2009) in general self-esteem. In line with oestrus in other species, it is possible that hormonal changes are more specifically connected to changes in directly mating-related constructs such as sexual motivation or attractiveness, but not general self-esteem. Additionally, it has been speculated that ovulatory changes are associated with reduced self-esteem to simultaneously promote women's mate-value enhancement when mating efforts are most critical (Hill & Durante, 2009). Given these conflicting results and the small number of studies, whether and how women's self-esteem varies across the cycle remains largely unclear.

Lastly, another aspect that is connected to both self-perceived attractiveness and self-esteem (Brown & Mankowski, 1993; Datta Gupta et al., 2016), but shows inconsistent changes across a woman's ovulatory cycle, is positive mood. Although findings on changes in mood across the cycle are generally mixed (Romans et al., 2012), most studies focus on mood as a part of premenstrual symptoms (Bäckström et al., 1983; Tschudin et al., 2010). There are fewer studies focusing on changes in positive mood across the whole cycle or specifically addressing ovulatory changes (Almagor & Ben-Porath, 1991). Among these, studies using daily self-reports show no differences in positive mood between different cycle phases (Almagor & Ben-Porath, 1991; Wilcoxon et al., 1976).

In conclusion, there is no clear picture of whether women's self-perceived attractiveness and desirability change across the ovulatory cycle and whether there exist ovulatory cycle shifts in related self-perceptions such as self-reported grooming behaviour, general self-esteem and positive mood. Previous ovulatory cycle research probably suffered from methodological problems such as incorrectly using between-subject designs for investigating within-subject effects, using a discrete instead of a continuous fertility estimator and low statistical power that can inflate type 1 error rates and false-positive findings (Gangestad et al., 2016).

We aimed to address this by conducting a preregistered and highly powered diary study comparing naturally cycling women with women using hormonal contraceptives. By investigating several attractiveness-related outcomes at the same time, this study also provides an insight into the different magnitudes of ovulatory cycle shifts. We predicted ovulatory increases in self-perceived attractiveness, desirability and grooming that are only present in the group of women not taking hormonal contraceptives. Based on the assumption that ovulatory changes are phylogenetically rooted in the oestrus that is observed in many other species, we expected ovulatory changes to be much stronger in mating-related self-perceptions. We expected no ovulatory increases in the broader domains of general self-esteem and positive mood. Our aim with this paper is to give an empirical overview of possible ovulatory changes in attractiveness-related self-perceptions in the same sample. As our data were observational, we do not aim to uncover associations between the different outcomes nor to imply a certain causal graph. We preregistered our study design, sampling methods, stopping rule and exclusion criteria as well as analytical steps. A detailed overview of all deviations from our preregistration that were necessary to refrain from falsely implying causality is shown under Table S1 in the Supporting Information.

## Methods

Since ovulatory cycle shifts are intraindividual changes, we used an online diary design as the appropriate assessment method for within-subject effects (Blake et al., 2016; Schmalenberger et al., 2021). This online diary is the second Goettingen Ovulatory Cycle Diary Study and was implemented using the online survey framework formr (Arslan, Walther & Tata, 2020). This framework enabled the complexity of the study design and also the automation of study parts with sensitive information to establish the anonymity of participants. All materials are accessible online, including survey files, data cleaning and codebooks (Arslan, Driebe et al., 2020, see also https://osf.io/d3avf/); the relevant analysis code for the study can be found at https://osf.io/2g4rc/. Owing to the intimate nature of data and because it cannot be fully anonymised, we will share data upon request.

### Recruitment and incentive structure

We recruited participants between May 2016 and January 2017 via a range of different digital strategies, such as social media (advertising via mailing lists of German university students and posting advertisements on okCupid.com, Facebook and on the study platform psytests.de), inviting eligible participants who had taken part in similar studies before and advertising the study in a first-year psychology lecture. Data collection ended in May 2017.

In order to compensate for the considerable effort of participation, the incentive structure was diverse. Participants received either a direct payment (between €25 and €45) or, alternatively, course credits for students of the University of Goettingen. All participants were given chances of winning lottery prizes with a total amount of €2000, and illustrated feedback on their own data. Prior to their involvement, participants were fully informed that their access to incentives depended on their participation rate and completion of the study.

### Procedure

After following an online study link, participants received detailed information about the study entitled ‘Everyday Life and Sexuality’, which was introduced as a study investigating the interaction of romantic relationships, sexuality and well-being. After providing their informed consent, participants answered the two initial surveys that assessed demographic and personality information. All personal and identifying data were collected and stored separately using formr features to further ensure anonymity.

The diary part began on the next day and encompassed a period of 70 consecutive days with daily self-reports. During this time, participants received email invitations and, if allowed, text message reminders with their personal study links every day at 5:00 p.m. Diary entries could be filled out until 3:00 a.m. the following morning. Daily questions asked for mood, health, daily activities and sexuality. If participants had already filled out a diary entry the day before, they were asked to rate the time between the last entry and the current one. If participants had skipped at least one entry beforehand, they were asked to rate the time spanning the previous 24 h. This method was used to cover the period of the diary continuously for users with high participation rates while avoiding responses where participants who had skipped entries would have aggregated across a much longer time than 24 h. To account for possible measurement reactivity biases (Arslan, Reitz et al., 2020), the order of the daily items was randomised within grouped blocks. As an additional strategy to facilitate high participation rate, the number of daily items was held low by applying a planned missing design: the probability of single items to be displayed on a specific day varied between 20 and 100% and for broader constructs with multiple items a subset of items was drawn randomly every day (see Table 1).

**Table 1.** Variables relevant to this study measured in the diary

Variable	Item (English translation)	Response format	Daily display probability (in%)
Onset of menstrual bleeding	‘Today was the first day of my menstrual bleeding …’	Yes No (yesterday) No (day before yesterday) No (3 days ago) No (4 days ago) No (5 days ago) No (6 days ago) No (onset longer ago)^a^	Once women indicated having menstrual bleeding on that day
Desirability	‘I felt sexually desirable’^b^	Five-point Likert scale: 0 (‘less than usual’) to 4 (‘more than usual’)	50
Attractiveness	‘I was satisfied with my appearance’ ‘I liked looking at myself in the mirror’ ‘I liked looking at my body’	Five-point Likert scale: 0 (‘less than usual’) to 4 (‘more than usual’)	30 30 30
Grooming	‘I was styled’ ‘I put effort into my outfit (clothes, make-up)’	Five-point Likert scale: 0 (‘less than usual’) to 4 (‘more than usual’)	30 30
Self-esteem	‘I was satisfied with myself’	Five-point Likert scale: 0 (‘less than usual’) to 4 (‘more than usual’)	80
Positive mood	‘My mood was good’	Five-point Likert scale: 0 (‘less than usual’) to 4 (‘more than usual’)	80

^a^Once women chose this option, a field appeared in which they could indicate the exact day of the onset of menstrual bleeding. ^b^Only women in a relationship were asked that question (66% of total sample).

**Figure 1.**
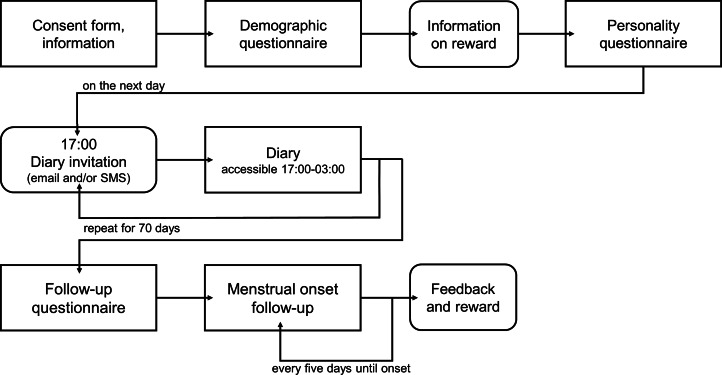
Overview of the study flow. The diary part spanned 70 consecutive days with one daily measurement.

After the diary, participants were asked to fill out three follow-up surveys: first, single participants answered a social network survey that is not part of the present study; second, all participants filled out a general follow-up survey assessing, among other questions, the use of hormonal medication and changes in contraception methods during the study; and third, those women who had not indicated menstrual bleeding within the last 5 days of the diary received an email invitation every 5 days to take part in the last follow-up survey that assessed the date of their next onset of menstrual bleeding. Following completion of the study, participants were fully debriefed and received personal feedback along with their respective compensations. A detailed overview of the study design is depicted in Figure 1.

### Measurements and variable transformations

#### Measurements

All variables of interest for the current study that were assessed in the diary part are shown in Table 1 with their corresponding response format and their display probabilities on each given day. Owing to an unfortunate coding error when designing the study, only women in heterosexual relationships were asked how sexually desirable they felt (66% of total sample, 355 women not using hormonal contraceptives, 221 women using hormonal contraceptives). All other variables were presented to the whole sample.

### Estimating women's fertility status

In order to obtain information about the ovulatory cycle during the diary, women were asked every 3 days, or after having skipped at least two consecutive diary entries, to indicate whether they had had menstrual bleeding during the previous 3 days or since their last diary entry, respectively. If they had, women were asked to report whether that entry day was the first day of menstrual bleeding or otherwise indicate the exact date of the onset (see Table 1). We also obtained the date of women's last onset of menstrual bleeding in the demographic survey at the beginning of the study as well as the date of their next onset of menstrual bleeding in the follow-up survey described above. Following Arslan, Schilling et al. (2018), we computed our main predictor of the ovulatory cycle using this information, the probability of being in the fertile window (PBFW), by backward counting from the next confirmed onset of menstrual bleeding. This method was recommended by Gangestad et al. (2016), who based their continuous PBFW estimates on Stirnemann et al. (2013). For this estimation, we only considered cycles that were between 20 and 40 days long and did not count further back than 40 days from the next onset of menstrual bleeding.

We preregistered that we would estimate women's fertility status with a method that was state-of-the-art at the time of analysis. By following the aforementioned recommendations, we believe we have adhered to this goal. In our preregistration, we also mentioned a procedure for averaging forward and backward counting methods to obtain a corresponding predictor. This procedure was necessary in previous studies with few observations of next menstrual onsets in order to avoid losing too many data points. However, in this study, sufficient information on next menstrual onsets could be collected. Therefore, we decided to refrain from averaging and use only the backward-counted PBFW, as recommended by Gangestad et al. (2016). Among other robustness analyses below, all models were re-run using an averaged PBFW predictor, yielding almost identical results (see Figure 4 and Figures S1–S4 in the Supporting Information).

Since using a continuous estimator across the cycle meant including menstrual or premenstrual days that might affect outcomes in ways unrelated to ovulation, we specifically coded these cycle phases and added them as control variables. To assess menstrual days, we asked women to report on every diary day whether they had had menstruation-related pain. Together with the information on menstrual bleeding described above and the resulting cycle length, this information was used to impute the probability of menstrual bleeding on each day. Additionally, the 6 days preceding the onset of menstrual bleeding were dummy-coded as the premenstrual phase.

### Exclusion criteria, participant flow and final sample

**Figure 2.**
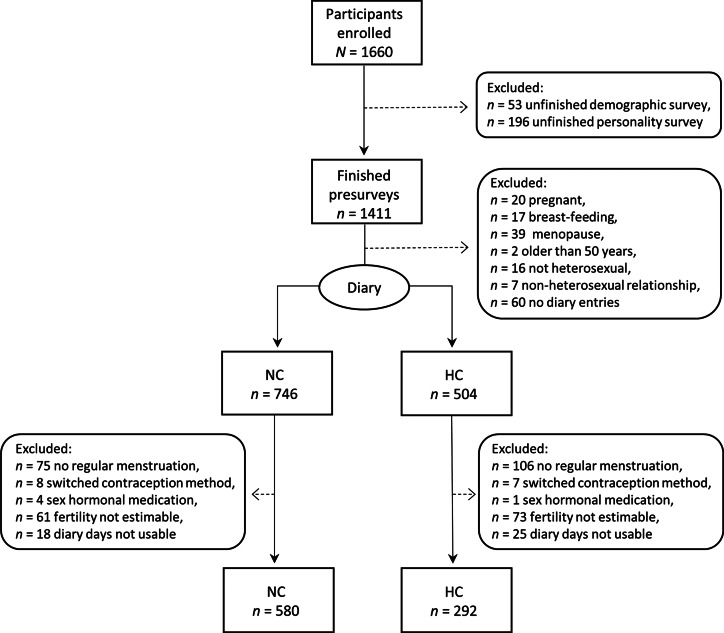
Participant flow and overview of exclusion criteria. If participants were affected by multiple exclusion criteria, only the first criterion is shown. NC, Naturally cycling women; HC, women using hormonal contraceptives.

Out of the total *N* = 1660 women who started the study, *n* = 1171 women completed the diary part and the general follow-up survey. As preregistered, we excluded women who did not take part in the diary and who were probably not experiencing ovulation, because of pregnancy, breast-feeding or menopause. Additionally, we sought to increase internal validity by excluding women whose ovulatory cycles might have been affected by taking sex hormones other than for contraception purposes or age above 50, or whose ovulatory cycles were irregular (those women who stated not experiencing menstruation ‘regularly (approximately monthly)’ in the demographic survey). Moreover, since we were interested in ovulatory cycle shifts in mating-related self-perceptions that presumably evolved to serve reproductive functions, women had to consider themselves predominantly heterosexual to be eligible for analyses. We also excluded unfinished diary entries and those where participants appeared to have been inattentive or dishonest. A detailed participant flow with the relevant exclusion criteria is depicted in Figure 2. The results of further robustness analyses using different exclusion criteria are discussed below and shown in Figure 4 and Figures S1–S4.

Consequently, our final sample consisted of *n* = 872 women, out of whom *n* = 580 (66.5%) were naturally cycling. In total, these women filled out 38,254 analysable diary entries with on average 43.9 (median 48, standard deviation, *SD*, 19.6) diary entries per woman. Participants were between 18 and 49 years old (mean, *M*, 25.5, *SD* 5.6), mostly students (66%) or employed (22%), held mostly Christian beliefs (49%) or were not religious (43%), and had on average 15.25 years of education (*SD* 4.72). On average, women's first menstrual bleeding occurred at the age of 12.7 (*SD* 1.3), their first sexual intercourse at the age of 17.0 (*SD* 2.8) and they had had 7.78 (*SD* 10.25) sexual partners. While 34% of women were single and 6% of women were in a non-committed relationship, 50% were in a committed relationship, 2% were engaged, 7% were married and 1% reported an undefined relationship status such as a temporary break-up. Seven per cent of women were mothers.

For non-hormonal contraception methods, most women (*n* = 258) used condoms only, *n* = 103 used fertility-awareness-based methods (with varying combinations with other non-hormonal methods), *n* = 53 used non-hormonal intra-uterine devices and *n* = 66 used other methods such as coitus interruptus (*n* = 12) or refraining from penetrative sex when fertile (*n* = 17). The remaining *n* = 100 women in the NC group reported not using contraception regularly.

For hormonal contraception, most (*n* = 153) women used the hormonal pill only, *n* = 96 used the hormonal pill combined with condoms and *n* = 29 used other hormonal contraception methods such as the vaginal ring. The remaining 14 women in the HC group used varying combinations of contraception methods, for example, hormonal pill, condoms and coitus interruptus (*n* = 2). Across the diary, the mean number of observed cycles was 2.52 (*SD* 0.84). The mean observed cycle length in the diary of 28.77 days (*SD* 3.07) matched closely the mean cycle length that participants had reported for themselves in the demographic survey at the beginning (*M* 28.52, *SD* 2.95).

As depicted in Table 2, HC and NC women differed from each other in some demographic variables, with the most important one being that HC women were on average nearly 3 years younger than NC women. Additionally, HC women had had fewer sexual partners and were more satisfied in their relationships. Possibly owing to self-selection for choosing contraception methods, HC women were more conscientious and less open to experiences, as measured with the Big Five Inventory (John et al., 1991). Concerning cycle characteristics, HC women had more regular ovulatory cycles and these were on average one day shorter, which might be a consequence of hormonal contraceptive use. Conducting a probit regression including the demographic variables in Table 2 except for the cycle characteristics, only age and number of lifetime sexual partners remained significant predictors of hormonal contraceptive use (*p* < .05). Besides these aspects, HC and NC women did not differ in their living situations, self-reported health, weight, weekly sport or weekly alcohol consumption.

**Table 2.** Descriptive statistics according to hormonal contraceptive use

	Mean (standard deviation)			
Variable	HC women	NC women	Hedges’*g*	*p*
**Age**	23.66 (4.43)	26.35 (5.86)	−0.46	<.001
Age at first sexual intercourse	16.79 (2.59)	17.09 (2.85)	−0.10	.133
Age at menarche	12.72 (1.26)	12.75 (1.38)	−0.02	.742
Relationship duration	3.4 (3.19)	4.16 (4.9)	−0.15	.025
Relationship satisfaction (0–5)	4.17 (0.76)	3.89 (0.9)	0.31	<.001
**Number sexual partners**	5.85 (8.65)	8.75 (10.88)	−0.27	<.001
Education years	14.89 (4.2)	15.43 (4.95)	−0.11	.089
Religiosity (0–5)	2.22 (1.36)	2.24 (1.35)	−0.01	.733
Cycle length	27.7 (2.34)	28.94 (3.14)	−0.39	<.001
BFI-Openness	3.72 (0.61)	3.82 (0.61)	−0.16	.015
BFI-Conscientiousness	3.63 (0.68)	3.48 (0.65)	0.23	.002
BFI-Extraversion	3.47 (0.82)	3.41 (0.76)	0.09	.195
BFI-Agreeableness	3.74 (0.62)	3.66 (0.59)	0.13	.059
BFI-Neuroticism	2.96 (0.78)	2.99 (0.77)	−0.04	.645

Note: NC, naturally cycling women; HC, women using hormonal contraceptives; BFI, Big Five Inventory. Variables are printed in bold if they remained significant after multivariate adjustment in a probit regression.

### Analyses

All analyses were performed using the statistical software R 4.0.2 (R Core Team, 2020) and the respective R packages lme4 (Bates et al., 2015) and lmerTest (Kuznetsova et al., 2017).

For all models, the main predictor was PBFW by backward counting from the next menstrual onset. As using PBFW as a continuous predictor across all days of the cycle meant including days of the premenstrual phase and menstruation too, we controlled for these variables by adding these phases as additional predictors to our models. Following Arslan, Schilling et al. (2018), we analysed the whole sample and used HC women as a quasi-control group to distinguish changes related to ovulation from other mid-cycle changes. Since most women taking hormonal contraceptives experience no ovulation but do have regular vaginal bleeding, comparing both groups helped ensure the ovulatory nature of these cycle shifts. Consequently, we included hormonal contraceptive use as a dummy variable (set to zero for NC women). To properly include interaction controls (Rohrer & Arslan, 2021), we amended our analysis plan in the preregistration with the interaction of hormonal contraceptive use with all predictors, not only PBFW. This decision was taken as the most appropriate modelling decision and not based on any result patterns. Among other robustness analyses such as using other exclusion criteria and fertility estimators as described below, we also ran models without interaction controls for premenstrual phase and menstruation. As can be seen in Figure 4 and Figures S1–S4, these analyses show no differences between the two modelling decisions. As preregistered, for all models we included random intercepts and random slopes for our main predictor variable PBFW. In Wilkinson's notation (Wilkinson & Rogers, 1973), our main models were specified as follows:
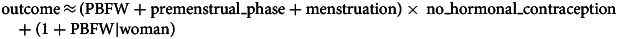


## Results

Adhering to our preregistration, we set the significance level to .01 to adjust for multiple comparisons. An extended overview of all linear mixed model results of our analyses is given in Table 3. We only report unstandardised effect sizes since all variables of interest were measured on commensurable scales and standardisation across different residual standard deviations might hinder comparability. Standardised effect sizes are shown in the robustness analyses in Figure 4 and Figures S4–S5, but differences from unstandardised effect sizes are small.

**Table 3.** Results of linear mixed effects models showing associations of cycle characteristics and women's self-perceptions

	Attractiveness		Sexual desirability		Grooming		Self-esteem		Positive mood	
Predictors	Estimate	99% CI	Estimate	99% CI	Estimate	99% CI	Estimate	99% CI	Estimate	99% CI
Intercept	1.84	1.79, 1.90	1.73	1.64, 1.81	1.62	1.56,1.68	2.10	2.04,2.15	2.16	2.10,2.21
PBFW	0.25	0.13, 0.36	0.38	0.17, 0.59	0.15	−0.00,0.30	0.13	0.02,0.25	0.13	0.01,0.26
Premenstruation	−0.04	−0.09, 0.00	−0.05	−0.14, 0.03	−0.04	−0.11,0.03	−0.03	−0.07,0.02	−0.02	−0.07,0.03
Menstruation	−0.02	−0.08, 0.03	−0.14	−0.24, −0.03	0.03	−0.05,0.10	−0.05	−0.10,0.00	−0.03	−0.08,0.03
HC (yes)	0.06	−0.04, 0.17	0.04	−0.11, 0.19	−0.00	−0.11,0.11	0.01	−0.09,0.11	0.04	−0.06,0.14
PBFW:HC	−0.38	−0.60, −0.16	−0.29	−0.65, 0.06	−0.25	−0.53,0.03	−0.21	−0.43,−0.00	−0.18	−0.40,0.05
Premens:HC	−0.00	−0.09, 0.09	0.05	−0.09, 0.20	0.04	−0.09,0.16	0.05	−0.04,0.13	0.04	−0.06,0.13
Mens:HC	−0.00	−0.11, 0.10	0.05	−0.12, 0.22	0.01	−0.14,0.15	0.06	−0.04,0.16	0.01	−0.10,0.12
**Random effects**										
*σ*^2^	0.70		0.99		1.06		0.77		0.92	
*τ*_00_	0.16_woman_		0.19_woman_		0.10_woman_		0.16_woman_		0.13_woman_	
τ_11_	0.26_woman.fertile_		0.40_woman.fertile_		0.14_woman.fertile_		0.28_woman.fertile_		0.26_woman.fertile_	
*ρ*_01_	−0.25_woman_		−0.37_woman_		−0.27_woman_		−0.28_woman_		−0.25_woman_	
*N*	868_woman_		568_woman_		865_woman_		870_woman_		869_woman_	
Observations	25,187		12,285		19,483		30,563		30,641	
Marginal *R*^2^/conditional *R*^2^	0.003/0.192		0.006/0.161		0.001/0.089		0.001/0.172		0.001/0.124	

Notes: PBFW, probability of being in the fertile window; Premens(trual phase), dummy-coded whether women are within 6 days preceding their onset of menstrual bleeding (0 = false, 1 = true); Mens(truation), estimated probability of women having menstrual bleeding; HC, dummy-coded whether women use hormonal contraceptives or not (0 = false, 1 = true); CI, confidence interval. Estimates represent unstandardised regression coefficients. ICC, Intraclass correlation; σ^2^, residual variance; τ_00_, between-subject variance; τ_11_, variance random slope; ρ_01_, correlation random intercept and random slope.

### Attractiveness

We found ovulatory increases in self-perceived attractiveness for NC women. Analysing 25,187 observations, self-ratings of attractiveness rose significantly with increasing PBFW (*b* = 0.25, *t*(1132.65) = 5.3, *p* < .001, 99% CI [0.13, 0.36]). This increase was significantly diminished in the group of HC women (*b* = –0.38, *t*(1320.92) = –4.42, *p* < .001, 99% CI [–0.60, –0.16]).

### Sexual desirability

We found ovulatory increases in self-perceived sexual desirability for NC women. Analysing 12,285 observations, self-ratings of sexual desirability rose significantly with increasing PBFW (*b* = 0.38, *t*(810.07) = 4.64, *p* < .001, 99% CI [0.17, 0.59]). This increase was descriptively diminished in the group of HC women (*b* = –0.29, *t*(886.70) = –2.15, *p* = .031, 99% CI [–0.65, 0.06]), but not significant according to our preregistered criterion. While not part of our predictions, we also found that sexual desirability significantly decreased with higher probability of menstrual bleeding in NC women (*b* = –0.14, *t*(11930.57) = –3.45, *p* < .001, 99% CI [–0.24, –0.03]). However, since we held no prior expectations regarding this finding, it should be interpreted with caution.

### Grooming

We found no significant ovulatory changes in self-reported grooming for NC women. Analysing 19,483 observations, self-ratings of grooming descriptively rose with increasing PBFW (*b* = 0.15, *t*(1357.87) = 2.52, *p* = .012, 99% CI [–0.00, 0.30]). This increase was descriptively diminished in the group of HC (*b* = –0.25, *t*(1506.40) = –2.29, *p* = .022, 99% CI [–0.53, –0.03]). Neither change was significant according to our preregistered criterion, but the confidence intervals may still include previously reported estimates.

### Self-esteem

We found ovulatory increases in self-esteem for NC women. Analysing 30,563 observations, self-esteem rose significantly with increasing PBFW (*b* = 0.13, *t*(1162.24) = 2.97, *p* = .003, 99% CI [0.02, 0.25]). This increase was significantly diminished in the group of HC women (*b* = –0.21, *t*(1303.80) = –2.59, *p* = .01, 99% CI [–0.43, –0.00]).

### Positive mood

We found ovulatory increases in positive mood for NC women. Analysing 30,641 observations, self-reported positive mood rose significantly with increasing PBFW (*b* = 0.13, *t*(1174.20) = 2.78, *p* = .005, 99% CI [0.01, 0.26]). This increase was descriptively diminished in the group of HC women (*b* = –0.17, *t*(1279.09) = –2.05, *p* = .041, 99% CI [–0.40, 0.05]), but not significant according to our criterion.

**Figure 3.**
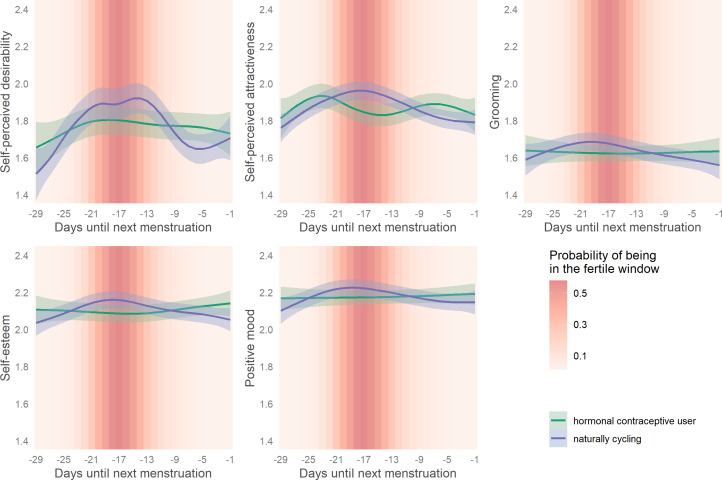
Changes in women's attractiveness-related self-perceptions across their ovulatory cycles. Smoothed curves calculated by generalised additive models using cyclic cubic splines. Days until next menstruation depict reverse cycle days backward counted from the next confirmed onset of menstrual bleeding. Bands represent 99% confidence intervals.

When plotting a smoothed spline over reverse cycle days, all outcomes showed small to moderate ovulatory increases as depicted in Figure 3.

### Robustness analyses

We conducted preregistered robustness analyses and further supplementary analyses to gauge the robustness of our results. We tested how various exclusion criteria affected our outcomes, probed our results for different estimates of fertility and compared different model specifications.

Regarding alternative exclusion criteria, we tested (1) no exclusions besides those necessary for estimating PBFW, (2) additionally excluding women who guessed that the study investigated fertile window effects, (3) excluding women who used any psychopharmacological, hormonal or antibiotic medication, (4) excluding women who were cycle-aware, (5a) excluding women who reported cycles with more than 2 days’ variability in length, (5b) excluding women who reported average cycle lengths shorter than 25 or longer than 35 days, (5c) excluding cycles shorter than 25 days in the diary, (5d) excluding women who were uncertain about the length and regularity of their ovulatory cycles, (6) excluding women who were trying to become pregnant, (7) excluding women who reported feeling unhealthy, (8a) including only women aged 18–25 years, (8b) including only women 26 years and older, (9a) including only Fridays to Sundays, (9b) including only Mondays to Thursdays, (10a) including only singles and (10b) including only partnered women. As an alternative method of estimating PBFW, we tested (1) not adjusting for (pre-)menstruation, (2) not adjusting for the interaction between hormonal contraception and (pre-)menstruation, (3) using forward-counting from the last menstrual onset, (4) averaging forward and backward counting estimates, (5) ‘squishing’ the follicular phase to a standard length before estimating PBFW, (6) counting backwards from the next menstrual onset inferred from the reported average cycle length, (7) using a discrete fertile window predictor when forward counting and (8) using a discrete predictor when backward counting. Regarding modelling choices, we (1) added varying slopes for the menstruation and premenstruation predictors, (2) added varying slopes but assumed them to be uncorrelated, (3) omitted varying slopes for PBFW, (4) required that the outcome have variance for each participant, (5) also report standardised effect sizes, (6a) adjusted outcomes for all other outcomes, (6b) adjusted for self-esteem, (6c) adjusted effects on self-esteem for mood and (6d) adjusted effects on desirability for grooming.

**Figure 4.**
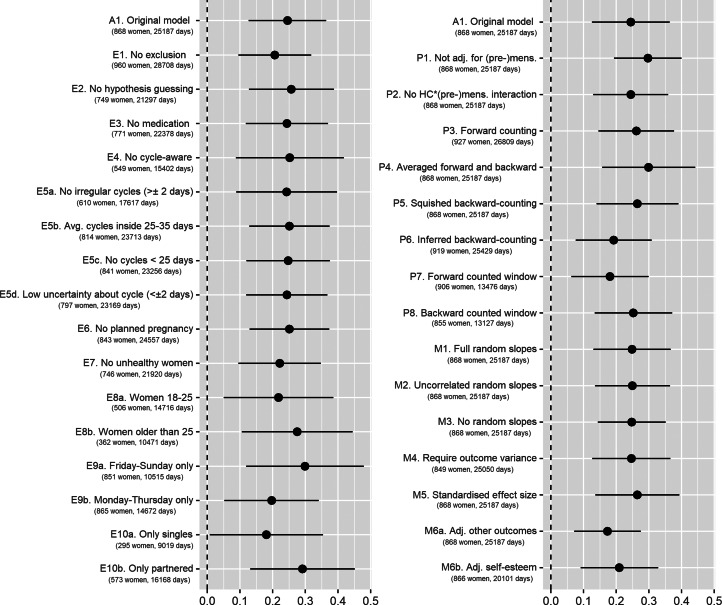
Effect of probability of being in the fertile window on self-perceived attractiveness with 99% confidence interval. A1 is the model described in the results section. Models starting with E are robustness analyses with different exclusion criteria. Models starting with P are robustness analyses with different specifications of the fertility predictor. Models starting with M are robustness analyses with different model specifications. Avg., Average; Adj., adjusted; HC, hormonal contraception; (pre-)mens, premenstrual and menstrual phase.

In the following, we seek to give a brief summary of these results. Importantly, for all models and robustness analyses, effects of PBFW differed in absolute size, but were rarely zero and never changed direction. A complete report of all these analyses including other visualisation methods and ordinal regressions showing the same result patterns can be found online (https://osf.io/2g4rc/). An overview of the conducted robustness analyses on attractiveness is given in Figure 4 and for the other outcomes in Figures S1–S4.

Regarding both attractiveness and sexual desirability, the results were largely robust. The significance of results was maintained in nearly all analyses and effect sizes varied only minimally. The sizes of PBFW effects on attractiveness peaked on weekends (*b* = 0.30, 99% CI [0.12, 0.48]) and in women in relationships (*b* = 0.29, 99% CI [0.13, 0.45]). The effect for sexual desirability peaked in women with low cycle irregularities (below 2 days, *b* = 0.46, 99% CI [0.19, 0.73]). Moreover, results were robust against adjusting for all other variables.

However, results for grooming, self-esteem and positive mood were less consistent. For grooming, most robustness analyses yielded non-significant cycle shifts, with some exceptions. A significant effect of PBFW emerged for example when only looking at women in relationships (*b* = 0.24, 99% CI [0.04, 0.44]) compared with single women, where the effect was the lowest (*b* = 0.024, 99% CI [–0.21, 0.26]). PBFW also became a significant predictor of grooming when using less valid methods for modelling the fertility estimate, such as forward counting to determine day of ovulation, ignoring possible influences of premenstrual and menstrual phases and ignoring the random effect structure of mixed models. Overall, effect sizes were small and the majority of analyses yielded non-significant results.

The effects of PBFW on self-esteem were robust for most fertility estimates and model specifications. Yet, the ovulatory increase in self-esteem varied according to several exclusion criteria. For example, when looking only at singles it was not significant (*b* = 0.08, 99% CI [–0.09, 0.26]), whereas when looking at women who were cycle unaware (not using awareness-based contraception or cycle tracking apps) the effect peaked (*b* = 0.21, 99% CI [0.05, 0.36]). The slight majority of robustness analyses supported significantly positive effects for PBFW.

The ovulatory increase in positive mood was the effect that showed the least robustness. The effect of PBFW held both in effect size and significance when dropping any exclusion criteria (*b* = 0.12, 99% CI [0.00, 0.23]) and it peaked in women who were cycle unaware (*b* = 0.23, 99% CI [0.07, 0.39]). However, many analyses of sample characteristics led to non-significant results, such as only using data of regularly cycling women (*b* = 0.11, 99% CI [–0.05, 0.26]) or women with good self-reported health (*b* = 0.12, 99% CI [–0.01, 0.26]). Additionally, decisions concerning fertility estimates and model specifications resulted in inconsistent results as well, with the effect becoming non-significant when using forward-counting methods to determine a fertile window (*b* = 0.00, 99% CI [–0.12, 0.12]). Whereas effect sizes varied only minimally, less than half of the conducted robustness analyses yielded significant results.

## Discussion

The current study used a highly powered daily diary design to address the question whether and which attractiveness-related self-perceptions of women show ovulatory increases across their ovulatory cycles. In support of our hypotheses, by comparing NC with HC women and by conducting a variety of robustness analyses, we found statistically significant ovulatory increases in self-perceived attractiveness, sexual desirability, self-esteem and positive mood. The ovulatory increase in grooming was small and absent for HC women, but while confidence intervals might still include estimates of previous studies, it failed to reach our preregistered significance level of .01.

### Attractiveness and sexual desirability

The finding of the existence of ovulatory increases in self-perceived attractiveness and sexual desirability is in line with previous research on ovulatory cycle shifts (Arslan, Schilling et al., 2018; Haselton & Gangestad, 2006). This study expands the previous, methodologically diverse literature by adding further robust evidence that women feel both more attractive and sexually desirable when fertile.

Although feelings of attractiveness and sexual desirability are similar and sometimes treated as equivalent, our analyses support previous findings that they are distinct constructs (Wade, 2000). Comparing effect sizes, it becomes apparent that sexual desirability descriptively shows a greater ovulatory increase (*b* = 0.38) than attractiveness (*b* = 0.25), and this general picture held across robustness analyses. Whereas more research is needed to disentangle these constructs, as was shown by Wade (2000), it is likely that they mostly differ in their sexual motivational component which in return could explain these different effect sizes. Looking at current literature on ovulatory changes in general, the predominant finding is that women show increased sexual motivation when they are fertile (Arslan, Schilling et al., 2018; Bullivant et al., 2004; Jones et al., 2019; Roney & Simmons, 2013, 2016; Shirazi et al., 2019). While the nature and function of these shifts remain a matter of debate (Arslan, Schilling et al., 2018; Gangestad et al., 2005; Havliček et al., 2015; Pillsworth et al., 2004; Pillsworth & Haselton, 2006; Stern et al., 2019, 2020), one hypothesis that is gaining more attention and empirical support is the motivational priority shifts hypothesis (Roney, 2016; Roney & Simmons, 2013). According to this hypothesis, estradiol and progesterone act as a two-signal code that promotes mating effort during the fertile phase, when reproductive fitness benefits outweigh the costs (risking injury, sexually transmitted diseases and opportunity costs with regard to e.g. foraging and feeding). Thus, the main adaptive psychological effect of ovulatory hormonal changes might be a general increase in sexual motivation. It is possible that ovulatory increases in self-perceived sexual desirability and attractiveness follow this dominant change in sexual motivation in order to promote mating effort (Haselton & Gangestad, 2006) and adaptively affect strategic mating decisions and mate choice standards (Penke et al., 2008; Todd et al., 2007). As feeling sexually desirable has been predicted to be more specifically linked to sexual motivation than general self-perceived attractiveness (Wade, 2000), this might also explain why the increase in sexual desirability is higher descriptively.

Another explanation of our finding could be that the effect of sexual desirability is artificially higher because we accidentally only assessed it in partnered women. Yet, when comparing it with the effect size of attractiveness only in partnered women (*b* = 0.29), the cycle shift in sexual desirability is still more pronounced. Additionally, relationship status did not influence self-perceptions of attractiveness and sexual desirability in prior studies (Arslan, Schilling et al., 2018; Haselton & Gangestad, 2006; Schwarz & Hassebrauck, 2008). Therefore, we deem it unlikely that effect sizes of sexual desirability would deviate much for single women.

Considering comparisons of NC and HC women, the ovulatory increases in self-perceived attractiveness and sexual desirability were substantially diminished in HC women, which supports the hormonal basis and internal validity of these ovulatory cycle shifts. This difference only became statistically significant for attractiveness, not for sexual desirability, but power is presumably the best explanation. As sexual desirability was accidentally assessed only in partnered women, resulting in a 34% reduction of sample size, the subsequent cut in statistical power is the most plausible reason why the interaction effect failed to reach significance for sexual desirability.

### Grooming

Unexpectedly, we did not replicate previous findings that women report increased grooming when they are fertile. While, descriptively, the effect was in the expected direction, it did not reach our strict criterion of significance and showed considerable variation in our robustness analyses. Together with the diary study of Arslan, Schilling et al. (2018), this study is the second highly powered longitudinal investigation to report a null finding for cycle shifts in self-reported grooming.

However, the sensitivity of our analyses for this outcome was smaller than that for the other outcomes, as the items were displayed more infrequently in our planned missingness design. Given the small estimated effect size, we may still have achieved insufficient statistical power. It is possible that an ovulatory increase in grooming does exist but that it is very small and consequently needs even higher statistical power to be detected. That an ovulatory increase in grooming, if it exists, is truly small could explain previous heterogeneous results. Another reason might be that previous research showing ovulatory increases in grooming mainly focused on clothing choices (but see Arslan, Schilling et al., 2018). In this study, we did not measure clothing choice specifically but operationalised grooming in a broader sense by asking the degree of styling in general and the extent of effort put into the participant's outfit. Moreover, our assessments were based on self-reports and not on external ratings of photographs or illustrations as was the case in Durante et al. (2008) and Haselton et al. (2007).

Finally, drawing from our robustness analyses, an ovulatory increase in grooming was present for a subsample of women who were in a relationship despite the subsequent reduced number of observations. Future research should consider relationship status as a moderating factor. Relationship dynamics might play an important role for the emergence of increased grooming when women are fertile. For example, it might be that grooming is enhanced only if another person serves as a romantic goal that these efforts are directed to. More research is needed to investigate whether only certain aspects of grooming change across the cycle and whether these differ according to relationship status or the availability of potential sexual partners in general.

### Self-esteem

We found an unexpected ovulatory increase in self-esteem that was only present in NC women. This contradicts previous findings of no significant ovulatory changes (Arslan, Schilling et al., 2018) or even ovulatory decreases in self-esteem (Hill & Durante, 2009).

According to the sociometer theory (Leary & Baumeister, 2000), self-esteem is an affect-laden self-evaluation indicating one's relational worth. The related hierometer theory by Mahadevan et al. (2019) views self-esteem as an indicator of social status. Considering the importance of women's attractiveness in their intrasexual competition and intersexual selection (e.g. Buss, 1988, 1989), attractiveness is likely to be one such factor determining relational worth and social status. Supported by the contingency of self-esteem on self-perceived attractiveness and desirability in women (Bale & Archer, 2013; Brase & Guy, 2004; Connors & Casey, 2006; Penke & Denissen, 2008), it seems plausible that the ovulatory increases in self-perceived attractiveness and desirability in this sample coincide with an ovulatory increase in self-esteem. Although Hill and Durante (2009) also argue a positive relationship of self-esteem and self-perceived attractiveness, they did not assess ovulatory changes in self-perceived attractiveness. Thus, it remains unknown whether and how an ovulatory change in self-perceived attractiveness compared with the ovulatory decrease in self-esteem that they reported.

Besides clear methodological differences regarding higher sample size, longitudinal assessments and continuous fertility estimates in the present study, relationship status could also explain the discrepant results. Hill and Durante (2009) report that seeking long-term partners moderated the ovulatory cycle shift in self-esteem insofar as the ovulatory decrease in self-esteem was higher the more women were seeking long-term partners. While we did not measure women's wish for long-term partners, we found differences in the ovulatory cycle shift according to relationship status. For single women only, the ovulatory increase in self-esteem was not significant. Although relationship status showed no additional effect in Hill and Durante (2009), it might be that other, currently overlooked effects influence women's self-esteem across the cycle. It is possible that, assuming that women experience an increase in sexual motivation when fertile, mating effort and mate value become more salient. Consequently, it is a woman's evaluations of her mate value that affect her self-esteem, in line with the sociometer theory and hierometer theory. For example, a woman seeking a partner but not having one when her sexual motivation and salience of mate value increase might down-regulate her mating-related self-esteem, whereas a woman who wants to have sex and has the possibility to have it, might up-regulate her mating-related self-esteem. Given that Arslan, Schilling et al. (2018) investigated only women in relationships, the difference in results may be surprising. However, Arslan, Schilling et al. (2018) used a self-esteem item with more trait variation (an intraclass correclation (ICC) of approximately .42, compared with our ICC of .16). It is possible that their item was less sensitive to intra-individual changes than ours. The question of whether ovulatory changes in self-esteem are dependent on women's sexual motivation and self-perceived mate value poses a fruitful topic for future research.

### Positive mood

Although we based our prediction on studies using daily assessments that indicated no ovulatory changes in positive mood, there are also studies using daily assessments that support our unexpected finding that positive mood increases when women are fertile. For example, Rossi and Rossi (1977) combined forward and backward counting methods to define the fertile phase of 67 women across 40 days and reported a clear ovulatory peak of positive mood that was only present in NC women. However, using the same counting methods as Rossi and Rossi (1977), McFarlane et al. (1988) compared daily data for 60–70 days of 27 women (12 using hormonal contraceptives). They found increased pleasant mood that was absent in the ovulatory phase but present in the menstrual and follicular phase only for NC women. Taken together, even studies that used similar study designs and methods reached opposing conclusions. The current study addresses the problem of low sample sizes that might have previously accounted for these inconsistencies. However, the ovulatory increase in positive mood showed low robustness across modelling decisions and different sample characteristics. Since we believe that our modelling decisions are appropriate, this highlights the importance of sample characteristics and interindividual differences in the effect of the ovulatory cycle on mood (Metcalf et al., 1989; Walker, 1994).

Unlike Rossi and Rossi (1977), we found that the ovulatory increase was descriptively but not statistically different between NC and HC women. This is in line with previous research that found no differences in the cyclical changes of mood between NC and HC women (Marriott & Faragher, 1986). Hence, we cannot rule out the possibility that other mid-cycle changes unrelated to ovulation drive the effect of PBFW on positive mood.

### General discussion

Comparing the effect sizes and robustness analyses of the investigated ovulatory cycle shifts, we found the strongest ovulatory increase in women's self-perceived sexual desirability, followed by women's self-perceived attractiveness. Ovulatory increases in self-esteem and positive mood were smaller and less robust. Although the small effect size of ovulatory increases in grooming was comparable with those of self-esteem and mood, it did not reach our strict significance criterion.

However, we cannot confidently infer whether, for instance, self-esteem increased solely because women felt more sexually desirable. Although such questions are often hastily addressed by statistical control or mediation analyses, claiming causality for observational data depends on assumptions that we found difficult to justify (Rohrer, 2018; Rohrer et al., 2021). We added exploratory analyses to our robustness analyses, in which we adjusted for other measured outcomes. However, because outcomes were measured with varying amounts of error and covariates were often missing because of our planned missingness approach, these analyses should only be seen as a starting point for future research. To untangle the causal web of related ovulatory changes, we need different designs. Direct, physiological measures of women without make-up might help us find out whether ovulatory changes in, for instance, skin quality rather than grooming, explain the self-perceptions of desirability. Experience sampling might help us understand whether self-esteem changes follow self-perceptions of ovulatory increases in attractiveness.

Additionally, a theoretical approach is necessary that embeds these attractiveness-related ovulatory cycle shifts. It might be that the main function of cyclical hormonal fluctuations, especially of estradiol and progesterone, is calibrating the trade-off of mating and feeding efforts as suggested by the motivational priority shifts hypothesis. Consequently, it would be plausible to assume ovulatory increases in constructs that are associated with ovulatory increases in sexual motivation. Relative magnitudes of ovulatory cycle shifts in self-perceptions might reflect the strength of the association of these self-perceptions to sexual motivation. This is hinted at in our results, with the highest ovulatory increase being sexual desirability, followed by attractiveness and smaller increases in self-esteem and positive mood. Yet there are different theoretical approaches that try to account for the ovulatory increase in sexual motivation in women (Arslan, Schilling et al., 2018; Gangestad et al., 2005; Gildersleeve et al., 2014; Havliček et al., 2015; Pillsworth et al., 2004; Pillsworth & Haselton, 2006; Stern et al., 2019, 2020; Wood et al., 2014). In the face of these debates, there is a great need for methodologically sound studies, preferably using open science practices, before any final conclusions about the functions or associations of ovulatory cycle shifts can be drawn. Moreover, no current theoretical approach addresses the question whether and to what degree any ovulatory cycle shift might translate into biologically relevant outcomes, for example regarding women's mate choices or reproductive fitness. Besides more rigorous methods, a theoretical and empirical debate is called for that discusses the nature of the biological relevance of ovulatory cycle shifts (e.g. do increases in self-perceived attractiveness translate into a differential mate choice and affect relative number or viability of offspring?) and their smallest effect size of interest (e.g. which differences in mating decisions or partner mate value might be expected to have an impact on reproductive fitness?).

Another interesting topic for future research is whether other people also perceive any of these ovulatory cycle shifts in women. This could answer the question whether women's increased feelings of attractiveness follow internal states or are based on observable changes or even social feedback, for example from mating partners. In particular, many early studies reported that men perceive ovulatory changes in women's attractiveness as a possible cue to fertility (Bobst & Lobmaier, 2012; Cobey et al., 2013; Haselton & Gildersleeve, 2011; Roberts et al., 2004; Schwarz & Hassebrauck, 2008). However, more recent studies challenge this finding, for example by questioning whether postulated shifts in facial shape or colour exist or are even perceptible (Burriss et al., 2015; Catena et al., 2019). Whether shifts are perceptible has clear implications for theory. Shifts below a perceptible threshold could be more easily explained from the perspective that oestrus has been ‘lost’ or is even ‘hidden’ in humans. Future studies not only should try to answer these questions but should also expand them to see if ovulatory cycle shifts in self-perceived sexual desirability, self-esteem and positive mood are related to externally observable attractiveness changes across the cycle.

### Limitations

Biases such as social desirability and recall bias might have affected our results. By using an online diary study that implemented features to ensure anonymity and by asking participants to never recall more than the last 24 h, these biases are probably attenuated but cannot be ruled out.

Another limitation is our assessment of ovulatory timing and the fertile phase. Backward counting from the next onset of menstrual bleeding is the best practice for counting methods, but it is still outperformed by ultrasound or hormonal measurements, especially luteinising hormone tests (Gangestad et al., 2016). However, using these methods was not feasible for an online diary study of this size. Well-validated proxy variables like ours still enhance the statistical power of a design because of the larger affordable and reachable sample. Future research that uses biological markers of ovulation and combines them with a large sample size would be desirable.

Additionally, because of the complexity of our diary study we mostly used single-item measures to lessen the time and effort for participants. This probably promoted a higher sample size and reduced non-response bias but came at the cost of using less-established measurements. The general discussion of the practical use of single-items is ongoing (Arslan, Brümmer et al., 2020; Fisher et al., 2018). However, future studies that ideally build on overarching theoretical assumptions of the nature of ovulatory cycle shifts could focus more on specific outcomes and validate our findings with more established scales.

Importantly, like the majority of studies in this field, our sample mostly consisted of young, educated participants from a developed Western country. Thus, our sample fulfils all aspects of a WEIRD sample (Henrich et al., 2010) and generalisability to other cultural backgrounds is limited. We expect the functional hormonal basis of ovulatory cycle shifts to be universal among humans, but cycle shifts can be conditional on age, parity, nutritional condition and health state. More research is needed to support the claim of the universality of ovulatory cycle shifts across different cultures and investigate how they change according to different hormonal levels.

### Conclusion

In this large, preregistered online diary study across 70 consecutive days, we found ovulatory increases in women's self-perceived attractiveness, sexual desirability, self-esteem and positive mood. We did not confirm previous findings of increased self-reported grooming when women are fertile. Comparing NC with HC women, ovulatory increases were present only in NC women for attractiveness and self-esteem. Ovulatory increases in sexual desirability and positive mood differed descriptively but not significantly between NC and HC women. Thus, we cannot rule out that increases in sexual desirability and positive mood follow other, unrelated mid-cycle changes instead of being ovulatory. Previous studies largely were not preregistered, had low sample sizes, used discrete estimates of fertility instead of continuous ones and used between-subject designs to investigate within-subject effects. Together, these factors can inflate false positives and false negatives. Although this study addresses these shortcomings and provides more reliable results, it also shows heterogeneity in ovulatory changes according to sample characteristics and analytical decisions for grooming, self-esteem and positive mood. Not only is more research needed to account for these interindividual differences, but future studies should also address how the reported shifts are associated with each other and explain causal or directional influences between them. Most importantly, there is a need for a theoretical framework that embeds these attractiveness-related self-perceptions in a broader picture of the nature and function of ovulatory cycle shifts.

## Research transparency and reproducibility

All material of this study including survey files, data cleaning and codebooks are accessible at https://osf.io/d4avf/ and the respective analysis code for this study can be found at https://osf.io/2g4rc/. Owing to the intimate nature of data and because it cannot be fully anonymised, we will share data upon request.

## Supplementary material

For supplementary material accompanying this paper visit https://doi.org/10.1017/ehs.2021.44. click here to view supplementary material
