# Supplementary material for: Women feel more attractive before ovulation: evidence from a large-scale online diary study
Source: Evol Hum Sci. 2021 Sep 1;3:e47. doi: 10.1017/ehs.2021.44 (PMC10427307; doi:10.1017/ehs.2021.44)
Supplement: Supplementary file 1 [file S2513843X2100044Xsup001.docx]

# Supplemental Material for

**“Women feel more attractive before ovulation: evidence from a large-scale online diary study”**

Lara Schleifenbaum^1,2*^, Julie C. Driebe^1^, Tanja M. Gerlach^1,2^, Lars Penke^1,2^, Ruben C. Arslan^2,3,4^

^1^Georg August University Goettingen, Germany

^2^Leibniz ScienceCampus Primate Cognition

^3^University of Leipzig, Germany

^4^Max Planck Institute for Human Development, Berlin, Germany

*Correspondence regarding this article should be addressed to Lara Schleifenbaum,
Email: lara.schleifenbaum@uni-goettingen.de, Gosslerstrasse 14, 37073 Goettingen, Germany.

**Supplementary Table S1.**
*Preregistration Planning and Deviation Documentation (PPDD) for our preregistration found at https://osf.io/d3avf/.*

| Preregistered Approach | Deviation | Explanation | Might deviations change the pattern of results? |
| --- | --- | --- | --- |
| We preregistered the following hypotheses:   1. There are ovulatory increases in grooming 2. There are ovulatory increases in attractiveness (named vanity) 3. There are ovulatory increases in self-perceived desirability and these are over and above changes in grooming 4. There are no ovulatory changes in self-esteem and if there are, this change is independent of variations in daily mood | We did not test hypotheses c) and d) as preregistered.  For c), we omitted the last part and did not check whether the change in self-perceived desirability is above the change in grooming.  For d), we omitted the last part and did not check whether the change in self-esteem is independent of variations in mood. Instead, we decided to independently test our expectation that there are no ovulatory changes in positive mood. These changes in tested hypotheses were decided upon before any analyses were conducted. | Hypotheses c) and d) were formulated that way as an attempt to uncover associative and possibly causal patterns. However, we subsequently started to doubt that mediational analyses in our observational data would speak to causal patterns.  Instead, we formulated a simpler goal to give a general overview of ovulatory changes in women’s attractiveness and related constructs. We hope future research can conduct more targeted probes to find which ovulatory shifts are rather primary and which rather secondary. | As can be seen in Figure S1 below, controlling ovulatory changes in self-perceived desirability for grooming did not change any results.  Controlling ovulatory changes in self-esteem for positive mood (Figure S3) reduced effect sizes in both the fertility effect and the group comparison to hormonal contraceptive users. Moreover, *p*-values of both predictors lay above our preregistered significance level of .01. Ovulatory changes in self-esteem and positive mood do not seem to be independent, but it is not clear whether one causes the other. |
| In order to collect enough data of naturally cycling women we could analyse, we preregistered to pay women, who   - reported their gender as female (not “other”), - were younger than 50, - deemed themselves predominantly heteroexual, - were not pregnant or breastfeeding now or had been during the last three months, - were not actively trying to become pregnant, - were not using hormonal contraception or medication now or in the last three months, - were not using psychopharmacological medication - deemed themselves pre­menopausal - and who reported menstruating regularly at the moment   The criterium of not using hormonal contraception was only included here as part of our incentive structure, not as an exclusion criteria since we would obviously need these women as our preregistered quasi-control group. | We did not exclude women:   - who are actively trying to become pregnant - who are using psychopharmacological medication - who are using hormonal medication now or in the last three months | We did not preregister our exclusion criteria in sufficient detail (whether we would do so for main analyses or only in robustness checks). Thus, we used the set of exclusion criteria that seemed the most plausible to us but report robustness analyses with both stricter and less strict criteria. We did not exclude women who were trying to get pregnant, because we observed next menstrual onsets for all included women. We only excluded women taking sex hormones, and decided to exclude women using other hormonal medication (primarily, thyroxine) and psychopharmacological medication as a robustness check. | As can be seen in our robustness analyses under “E3. No medication”, additionally excluding women who used psychopharmacological, hormonal, or antibiotic medication did not change the results for the outcomes attractiveness, self-perceived desirability, grooming and self-esteem. Applying this additional set of exclusion criteria to the outcome positive mood did affect the results. However, as discussed above, this outcome shows the least robustness for any decisions concerning inclusion and analytical choices. |
| We preregistered that we would use multiple imputation or a similar feasible method for our planned missingness design. | We did not impute any data and only analysed complete observed data. | We found that multiple imputation for a multilevel model like ours was computationally challenging (i.e., more complex procedures often did not work as advertised), required many assumptions that we could not endorse (such as the absence of variation in slopes), and were unlikely to improve estimates when it was the outcome that was unobserved. | We conducted an in-depth investigation on one item (risk taking) to determine whether multiple imputation would affect standard errors and found no appreciable differences with the models we were able to compute (not shown). |
| We preregistered that we would use the package brms (Bürkner, 2017) for analyses using Bayesian inferences. | We used lme4 (Bates *et al.*, 2015) and lmerTest (Kuznetsova *et al.*, 2017) for general linear mixed models for all our analyses. | We changed the respective analytical method because of the expertise of the first author for frequentist statistical approaches. | Given that we did not preregister informative priors, the results can be expected to largely converge between Frequentist and Bayesian estimation. The last author confirmed this through several reanalyses (not shown). |
| Our preregistered analytical models included main effects for fertility, premenstrual phase and menstruation, an interaction of fertility*hormonal contraception and a random intercept and random slope of fertility per woman. | We added the interaction of premenstrual phase and menstruation with hormonal contraception to our preregistered models. | As we learned after writing our preregistration, applying these interaction controls is the most appropriate way of modelling our control variables (Rohrer and Arslan, 2020). | As depicted in our robustness checks under “P2. No HC*(pre)mens. interaction”, this deviation did not change any results. |
| We preregistered robustness analyses to check:   1. whether the results differ by contraceptive method, specifically by whether women are fertility ­aware (i.e. using a counting or temperature method or using a cycle tracking app) 2. whether results are specific to the outcome of interest or driven by more general changes (e.g. whether sexual desire increases go above and beyond any increases in self ­esteem) 3. whether the outcome visually peaks at the estimated day of ovulation when using a generalized additive model or a simpler model across days on the X axis 4. whether excluding various participants who are potentially less likely to ovulate affects the effect size estimate 5. whether the specification of the predictor matters (we will at least compare forward­ vs. backward­counting, continuous predictor versus window estimation) 6. whether not adjusting for menstruation matters (we predict that it does for some outcomes, e.g. in­pair sexual desire and sexual activity, self ­perceived desirability) 7. whether effect sizes are moderated by    1. age    2. weekday    3. self ­reported average cycle length    4. self ­reported cycle regularity    5. self­ reported certainty about the details of own menstrual cycle    6. self-reported health | We modelled g) with separate models instead of moderation analyses and we interpret the results of b) more cautiously because we do not think the causal inference can necessarily be made. | Similar to our preregistered hypotheses (see first row of this table), we now deem mediational analyses largely uninformative for the causal question of whether one effect “drives” another. For the moderator robustness checks in g), group sizes for some subgroups were very small. That is why we decided to report analyses with these groups excluded (rather than including a group variable as a moderator). | We do not expect any of these deviations to have an effect on our reported results.  Instead, by using analyses with exclusion rather than moderation for g), we could more properly estimate the effects of sample characteristics for partly small subgroups. |

**Supplementary Figure S1***Effect of probability of being in the fertile window on sexual desirability with 99 % confidence interval*

*
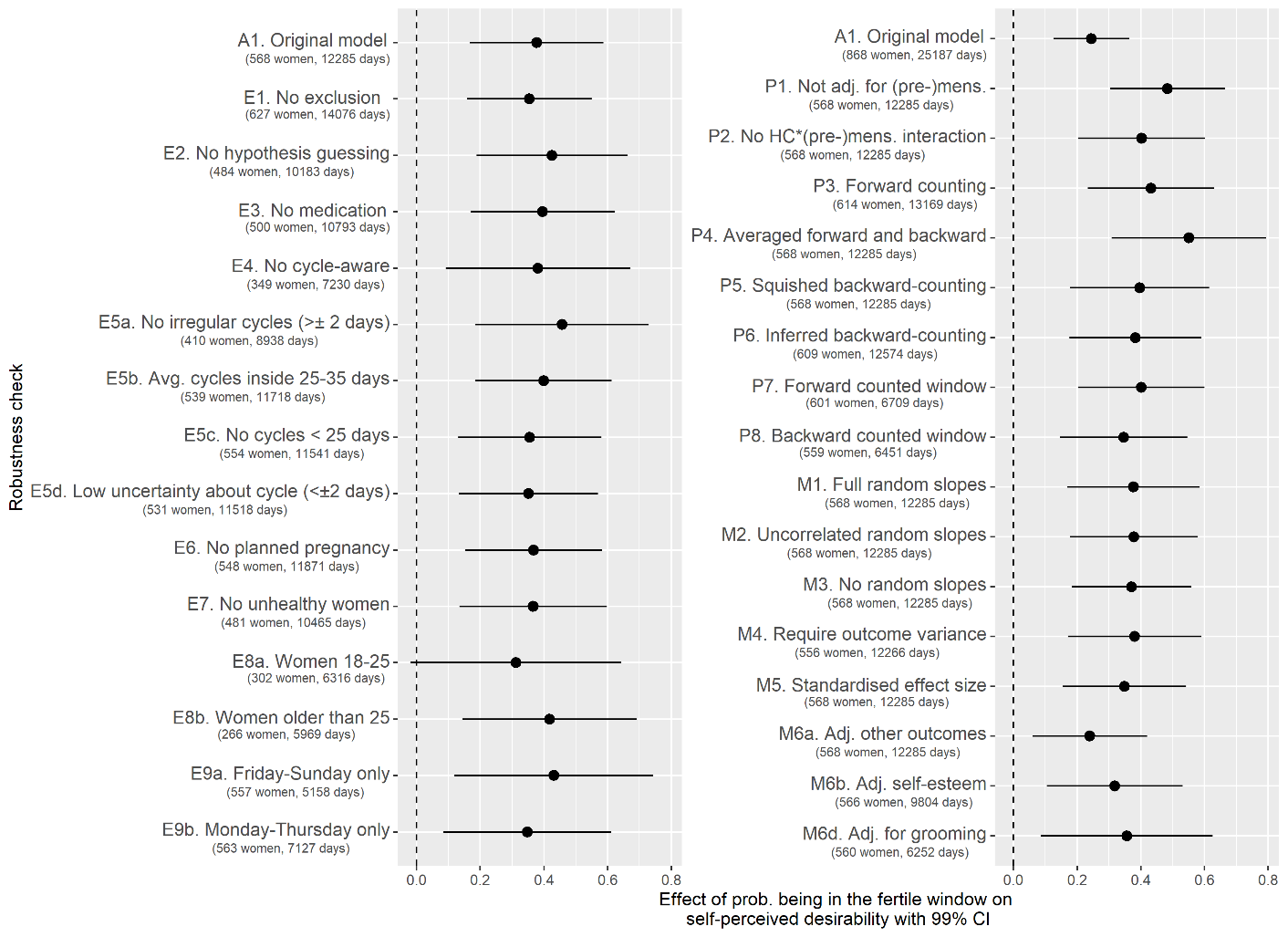
*

Note. A1 is the model described in the results section. Models starting with E are robustness analyses with different exclusion criteria. Models starting with P are robustness analyses with different specifications of the fertility predictor. Models starting with M are robustness analyses with different model specifications. Avg. = average, Adj. = adjusted, HC = hormonal contraception, (pre-)mens = premenstrual and menstrual phase.

**Supplementary Figure S2***Effect of probability of being in the fertile window on self-perceived grooming with 99 % confidence interval*


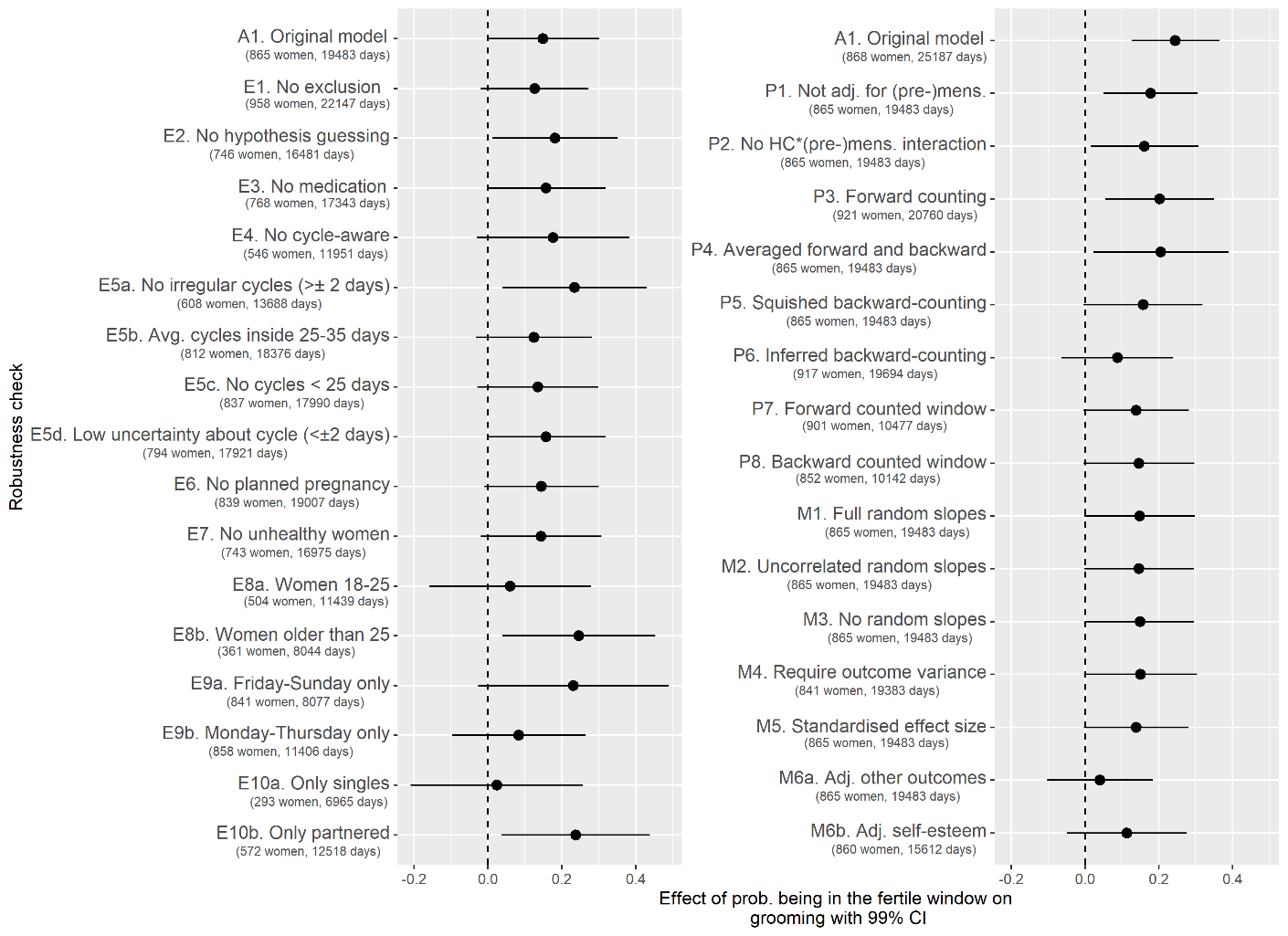
 Note. A1 is the model described in the results section. Models starting with E are robustness analyses with different exclusion criteria. Models starting with P are robustness analyses with different specifications of the fertility predictor. Models starting with M are robustness analyses with different model specifications. Avg. = average, Adj. = adjusted, HC = hormonal contraception, (pre-)mens = premenstrual and menstrual phase.

**Supplementary Figure S3***Effect of probability of being in the fertile window on self-esteem with 99 % confidence interval*

*
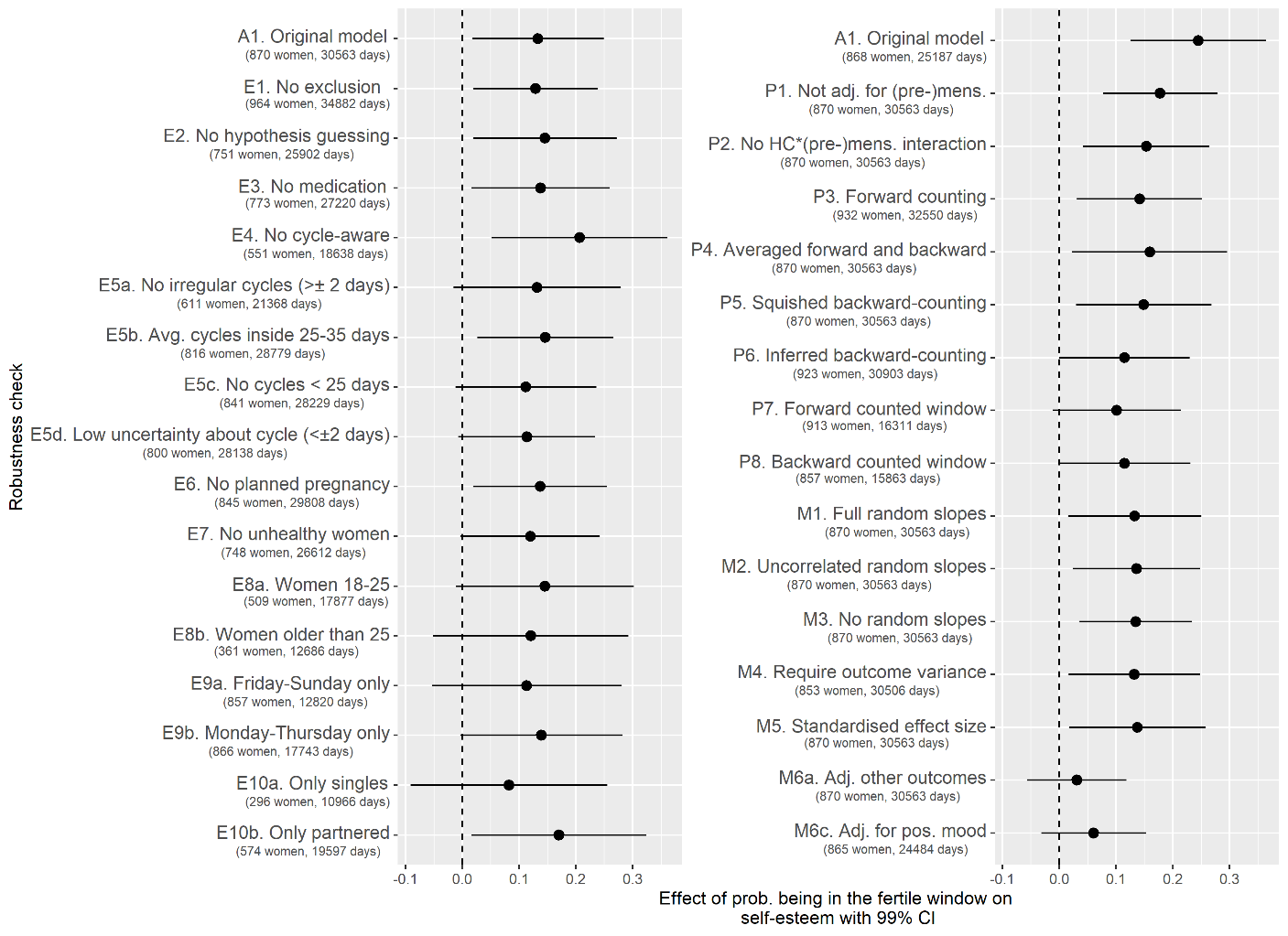
*

Note. A1 is the model described in the results section. Models starting with E are robustness analyses with different exclusion criteria. Models starting with P are robustness analyses with different specifications of the fertility predictor. Models starting with M are robustness analyses with different model specifications. Avg. = average, Adj. = adjusted, HC = hormonal contraception, (pre-)mens = premenstrual and menstrual phase.

**Supplementary Figure S4***Effect of probability of being in the fertile window on positive mood with 99 % confidence interval*

*
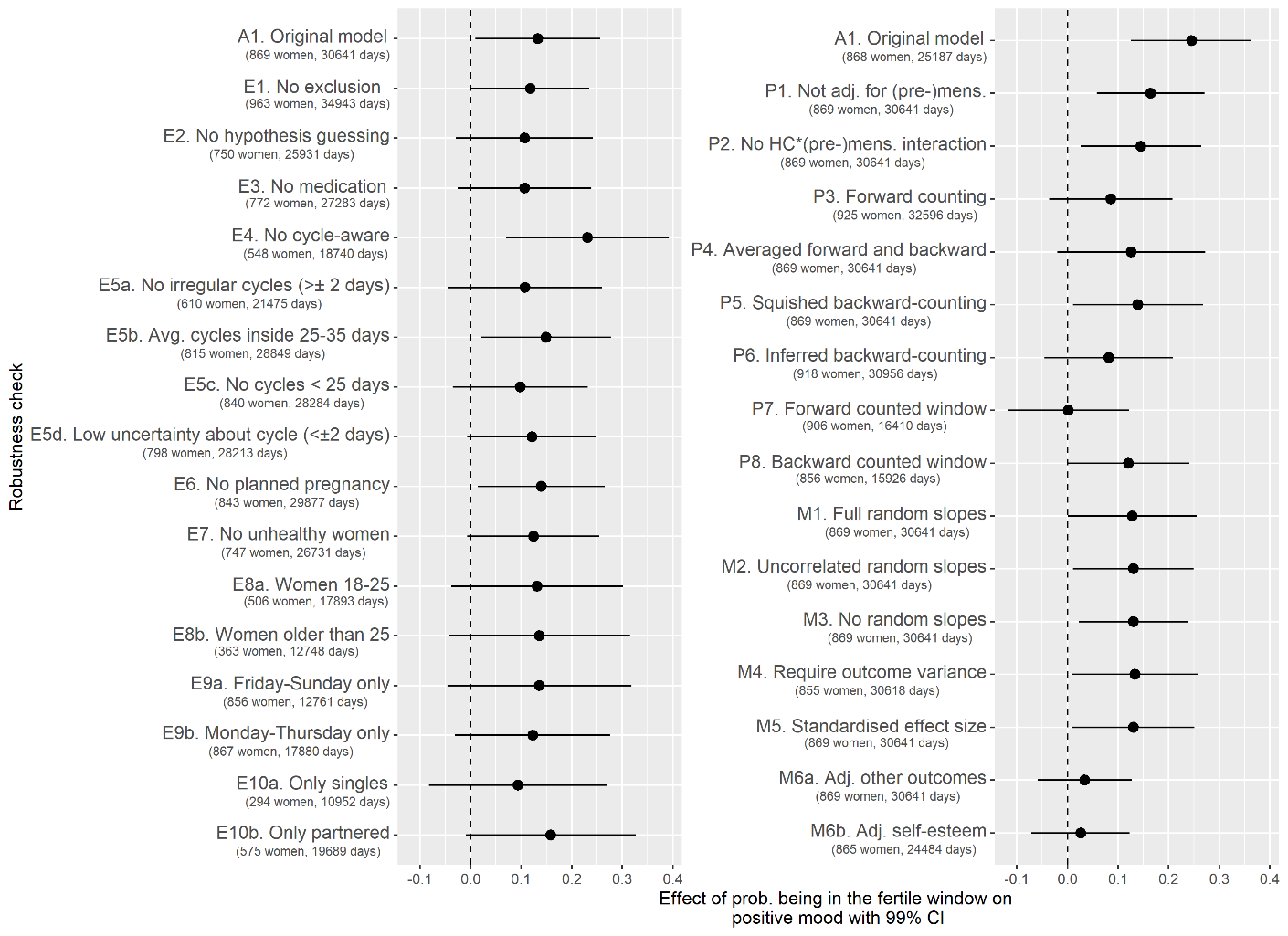
*

Note. A1 is the model described in the results section. Models starting with E are robustness analyses with different exclusion criteria. Models starting with P are robustness analyses with different specifications of the fertility predictor. Models starting with M are robustness analyses with different model specifications. Avg. = average, Adj. = adjusted, HC = hormonal contraception, (pre-)mens = premenstrual and menstrual phase.

References

Bates, D., Mächler, M., Bolker, B., & Walker, S. (2015). Fitting Linear Mixed-Effects Models Using lme4. *Journal of Statistical Software*, *67*(1). https://doi.org/10.18637/jss.v067.i01

Bürkner, P.‑C. (2017). brms : An R Package for Bayesian Multilevel Models Using Stan. *Journal of Statistical Software*, *80*(1). https://doi.org/10.18637/jss.v080.i01

Kuznetsova, A., Brockhoff, P. B., & Christensen, R. H. B. (2017). lmerTest Package: Tests in Linear Mixed Effects Models. *Journal of Statistical Software*, *82*(13). https://doi.org/10.18637/jss.v082.i13

Rohrer, J. M., & Arslan, R. C. (2020). Precise Answers to Vague Questions: Issues With Interactions. *PsyArXiv.* Advance online publication. https://doi.org/10.31234/osf.io/7fm2j
